# Multivariate Curve Resolution for 2D Solid-State NMR spectra

**DOI:** 10.1021/acs.analchem.9b05420

**Published:** 2020-02-18

**Authors:** Francesco Bruno, Roberto Francischello, Giovanni Bellomo, Lucia Gigli, Alessandra Flori, Luca Menichetti, Leonardo Tenori, Claudio Luchinat, Enrico Ravera

**Affiliations:** † Magnetic Resonance Center (CERM), University of Florence, and Consorzio Interuniversitario Risonanze Magnetiche di Metalloproteine (CIRMMP), via L. Sacconi 6, 50019 Sesto Fiorentino, Italy; ‡ Department of Chemistry “Ugo Schiff”, University of Florence, via della Lastruccia 3, 50019 Sesto Fiorentino, Italy; § Institute of Clinical Physiology, National Research Council, Via G. Moruzzi, 1 56124 Pisa, Italy; ∥ Dipartimento di Chimica e Chimica Industriale, Università di Pisa, Via G. Moruzzi 13, 56124 Pisa, Italy; ⊥ Fondazione Regione Toscana G. Monasterio, Via G. Moruzzi 1, Pisa 56124, Italy

## Abstract

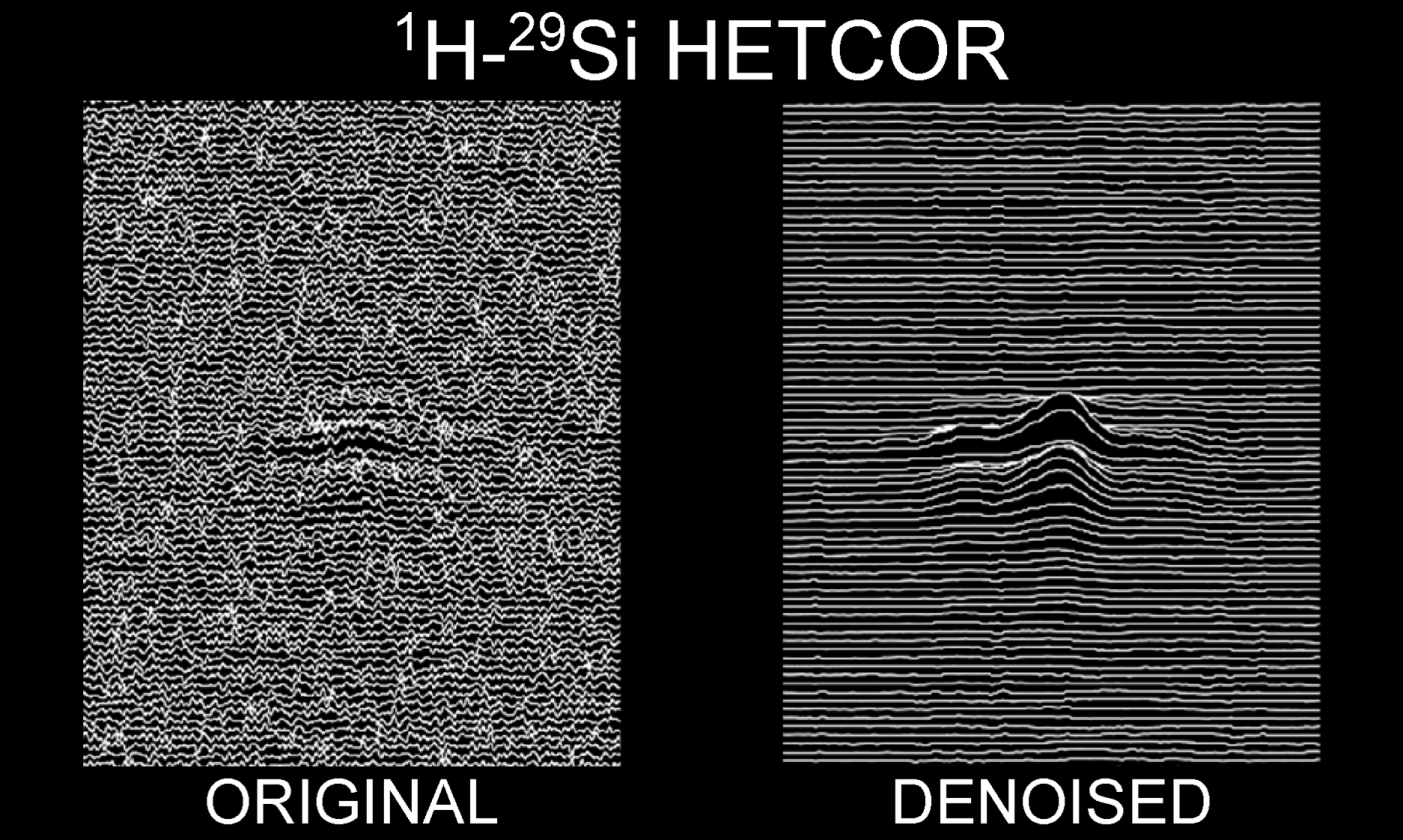

We present a processing method, based on the multivariate curve resolution approach (MCR), to denoise 2D solid-state NMR spectra, yielding a substantial S/N ratio increase while preserving the lineshapes and relative signal intensities. These spectral features are particularly important in the quantification of silicon species, where sensitivity is limited by the low natural abundance of the ^29^Si nuclei and by the dilution of the intrinsic protons of silica, but can be of interest also when dealing with other intermediate-to-low receptivity nuclei. This method also offers the possibility of coprocessing multiple 2D spectra that have the signals at the same frequencies but with different intensities (e.g.: as a result of a variation in the mixing time). The processing can be carried out on the time-domain data, thus preserving the possibility of applying further processing to the data. As a demonstration, we have applied Cadzow denoising on the MCR-processed FIDs, achieving a further increase in the S/N ratio and more effective denoising also on the transients at longer indirect evolution times. We have applied the combined denoising on a set of experimental data from a lysozyme–silica composite.

## Introduction

Sensitivity is one of the largest limitations in solid-state nuclear magnetic resonance (ssNMR) and becomes more severe as the gyromagnetic ratio and the natural abundance of the investigated nucleus decrease. Under these conditions, the signal intensity is low and, quite often, the low relaxation mechanisms efficiency requires experiments with long recovery delays. One relevant example is ^29^Si NMR. Silicon is the second most abundant element by mass on earth,^1^ has relevant functions in living organisms,^2^ and a large share of its compounds are solid.^3^^29^Si-solid-state NMR is, therefore, an election technique for a plethora of applications, but faces some silicon intrinsic limitations. ^29^Si can be described as a diluted isotope with medium sensitivity:^4^ the natural isotopic abundance is 4.7% and the frequency with respect to ^1^H is 19.9%; therefore, the receptivity of ^29^Si is roughly twice that of ^13^C in natural abundance. However, the overall sensitivity of ^29^Si NMR is often negatively impacted by the absence of nuclei with higher gyromagnetic ratios in the vicinity, rendering the relaxation times prohibitively long. This latter problem can be overcome by paramagnetic doping, at the price of altering the chemical composition of the sample.^5−8^

The low sensitivity, as is always the case in NMR,^9^ is due to the low Boltzmann population difference among the ground and excited states. One way to increase this difference is to increase the static magnetic field; commercial instruments with fields as high as 28.2 T (1.2 GHz ^1^H Larmor frequency) are now available.^10^ However, not only are high field magnets more expensive than low field magnets because of the different manufacturing processes, but their operational costs are also higher, so that, overall, the price per experiment gets significantly higher even when moving from 16.4 to 18.8 T (from 700 to 800 MHz). Another option is to use larger amounts of sample, but (a) the amount of sample is likely limited, (b) small rotors need to be used to increase the maximal achievable spinning speed and thus the resolution,^11^ and (c) more components may be present, imposing a strong dilution on the species of interest.^12,13^ Proton detection, available at high spinning frequency, can be used to increase the sensitivity,^11,14,15^ but this is necessarily limited to proton-rich materials. Dynamic nuclear polarization (DNP) is also a viable route to study silicon-based materials,^16−19^ but the equipment is more expensive than standard NMR. An interesting application of ultrafast acquisition based on gradient encoding has been proposed for high resolution magic-angle-spinning (MAS) of soft solids,^20,21^ but the applicability to rigid solids is also hardware-limited. Hence, methods based on data processing, rather than on data acquisition, might be welcome in preparatory studies, before the actual measurements performed at higher fields and/or using DNP. Consistent efforts are indeed devoted to the development of processing methods that allow for signal extraction from noisy spectra, in this and in different areas of NMR. These efforts led to several options to reduce noise: wavelet transform,^22^ Savitzky-Golay,^23^ random QR denoising,^24^ singular spectrum analysis,^25^ and Cadzow filtering.^26,27^ Each of these methodologies, however, has its own benefits and drawbacks.

We here propose for the first time the use of multivariate curve resolution (MCR) for denoising applications in ssNMR. MCR is a chemometric method primarily developed to recover pure components information from data of complex mixtures.^28−31^ Initially developed for UV–Vis spectroscopy,^32^ MCR has been then successfully applied to resolve data from a plethora of different analytical techniques,^33^ first of all chromatography, but also chemical reaction monitoring,^34^ spectroscopic imaging,^35^ environmental monitoring,^36^ and analysis of “omics” data sets, such as genomic^37^ and metabolomic data.^38^ It has also been applied to the deconvolution of 2D solution NMR data of reaction mixtures.^39^ MCR has the same aim as the blind-source-separation method, the applications of which to NMR are discussed in refs (40−43). We here show that this processing is extremely beneficial for ssNMR spectra. We also demonstrate that it can be also successfully applied to the simultaneous denoising of spectra of the same sample acquired at different mixing times, therefore offering the opportunity for also using the spectra with the lowest S/N ratio from a series.

Finally, while this method would be fully compatible with denoising the processed spectra,^39^ we have applied it for denoising time-domain data, thus preserving the possibility of applying further processing to the data. In particular, we have applied the Cadzow denoising to further increase the S/N ratio, as it appears to be well suited for ssNMR spectroscopy.^27^ This latter point turns out to be particularly relevant. Finally, through the whole process, quantitative information is preserved.

## Experimental Section

### Experimental Data

Solid-state NMR experiments were recorded on a Bruker Avance II spectrometer operating at 700 MHz ^1^H Larmor frequency (16.4 T), corresponding to 139 MHz ^29^Si Larmor frequency. The spectrometer is equipped with a 3.2 BVT MAS probehead in double resonance mode. The pulse lengths are 2.4 and 4.7 μs for ^1^H and ^29^Si, respectively. Cross-polarization was achieved by matching the *k* = 1 Hartmann–Hahn condition.^44^ The spectral windows for the different nuclei were 60 and 249 ppm for ^1^H and ^29^Si, respectively. During the ^1^H magnetization evolution under the chemical shift in the indirect dimension of heteronuclear correlation experiments, the PMLG decoupling sequence was used to suppress ^1^H–^1^H dipolar couplings.^45,46^ Spectra were acquired with CPMG echo train acquisition,^47^ and then the echoes were coadded.

### Synthetic Data

Synthetic data were generated over the same spectral window as the experimental data. The spectra in the series of three (see below) comprise of up to nine cross-peaks of variable intensities and line widths in the indirect dimension (SI Table S1).

### Data Processing

Indicating the features of the MCR algorithm according to the standard chemometric nomenclature, MCR decomposes the experimental data matrix *D* into a “Concentration” matrix *C* and a “Spectra” matrix *S*, and the part of the data that is not reproduced by the factorization contributes to the residual matrix *E* (see eq 2 in the Results and Discussion section). The factorization has been accomplished through alternating least-squares,^28^ starting from the purest variables^48^ estimate for the matrix *C* in the time domain. The number of components is set to 4, with 10% of the most intense signal as threshold for the noise.^28^ We have implemented the MCR algorithm in Python, but there are several implementations available in, for example, MATLAB.^49^ The operation is repeated until convergence, which is evaluated on the spectral norm of the difference between the matrices associated with two consecutive steps, and the optimization ends when the norm in any of the matrices goes below 10^–5^.

All spectra were processed using the NMR PIPE class from the NMRGlue library,^50^ with the parameters reported in SI Table S2.

The S/N ratio was estimated dividing the maximum signal intensity by the standard deviation of the noise, calculated as follows:
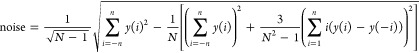
1where *N* is the total number of points in the noise region, *n* = (*N* – 1)/2 and *y*(*i*) is the *i*th point in the noise region. As a representative noise region, we selected a slice of the spectrum where no signal is present.

## Results and Discussion

### Application of the MCR Algorithm

According to the standard nomenclature used in chemometrics, MCR decomposes the data matrix *D* into a “concentration” matrix *C* and a “spectra” matrix *S*, leaving behind a residuals matrix *E*:
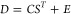
2

Intuitively, in a 2D NMR spectrum there is no variation in concentrations, but rather in the signal intensities because of indirect evolution. Therefore, the resulting matrix *C* contains the time evolutions of the indirect dimension, and the resulting matrix *S* contains the FIDs of the direct dimension. Given that we wanted to demonstrate that this method is applicable regardless of the line shape of the peaks, we limited the input of prior information to the analysis: no forward model (e.g.: using Gaussians for modeling the peaks), nor any regularization (e.g.: non-negativity of the spectra in the frequency domain, or smoothness), for either the FIDs or evolutions, were applied. Therefore, the factorization was obtained through a simple alternating least-squares approach:^28−30^ at the *k*th iteration, the values for *C*_*k*_ and *S*_*k*_ are obtained as
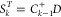

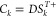
and
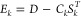
where “+” denotes the Moore-Penrose pseudoinverse. The initial guess for the indirect evolution matrix, *C*_0_, was obtained through the purest variables algorithm.^48^

We have used four components for the factorization, as this number ensures the best S/N with the least amount of bias and without increasing the number of iterations needed for optimization (see SI Table S3 for more details). Therefore, *S*^*T*^ is a complex matrix of dimension 4 × *N*, and *C* a complex matrix of dimension *M* × 4 where *N* and *M* are the number of points in the direct and indirect dimension respectively (see SI Table S2).

As already mentioned, a very important feature of MCR is that several spectra can be coprocessed with a common basis of FIDs (see below).

### Denoising on a Single Synthetic Spectrum

**Figure 1:**
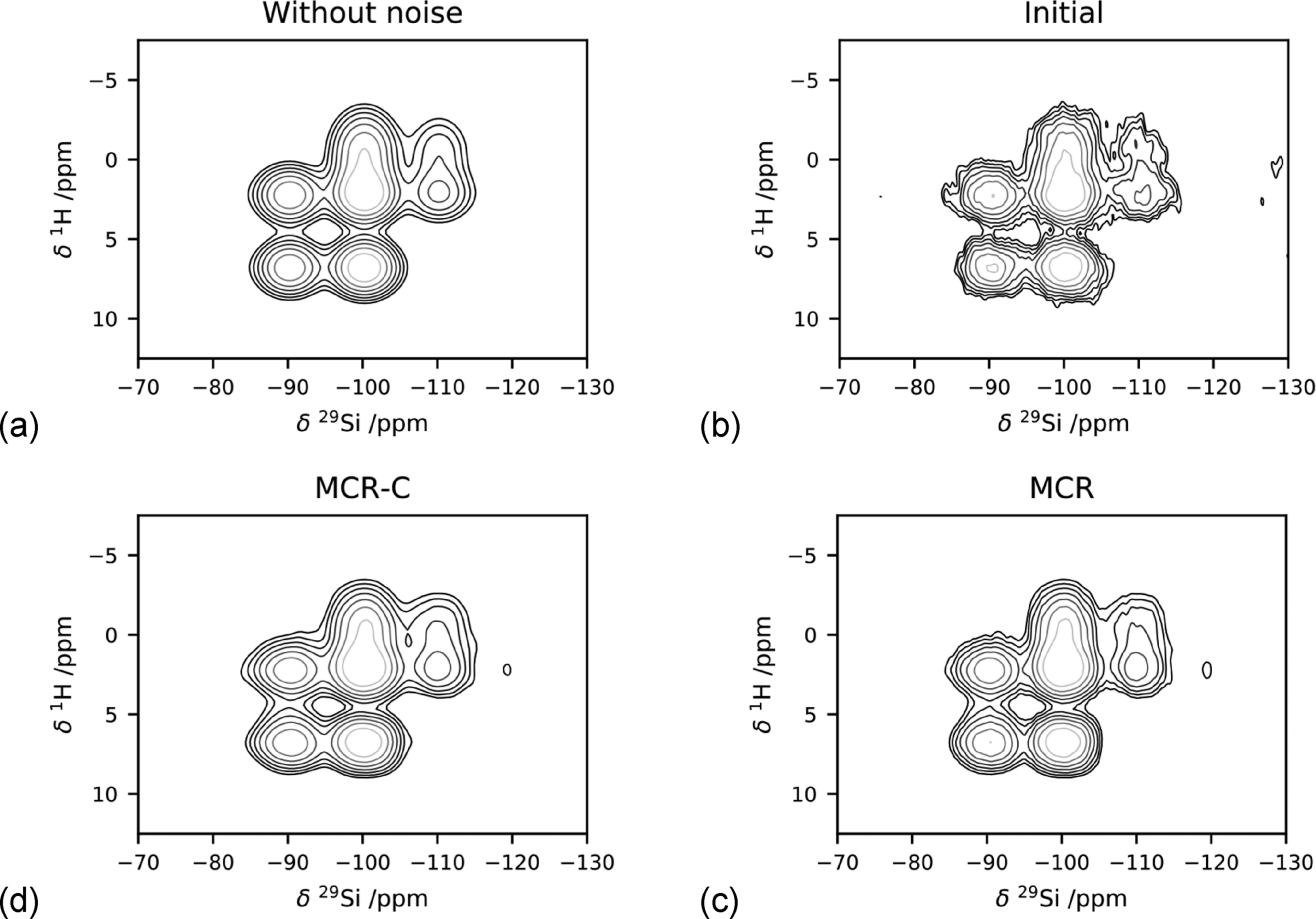
(a) Synthetic spectrum without noise and (b) synthetic spectrum with noise applied, (c-d) result of the processing of the (b) spectrum: (c) spectrum reconstructed with MCR and (d) spectrum reconstructed with MCR after application of Cadzow denoising.

The method was first tested on a single synthetic spectrum (Figure 1a). The peak positions, line widths, and intensities are given in Table 1. Gaussian noise was added to the spectrum (Figure 1b), yielding a S/N ratio of 46.

**Table 1:** S/N Ratio in the Different Spectra Across the Different Processing Steps

	spectrum					
step	A		B		C	
initial S/N	45		45		47	
	single	coprocessed	single	coprocessed	single	coprocessed
MCR S/N	106	206	221	150	250	153
MCR+Cadzow S/N	130	253	237	191	299	195

The denoising increases the S/N ratio to 221 after MCR and 237 after Cadzow denoising. Importantly, it does not alter the relative intensities of the peaks (SI Figure S1). To be noted that the Cadzow denoising gives more emphasis to the earlier points of the time domain and requires additional exponential apodization,^27^ therefore it imposes a modest line broadening to the reconstructed spectrum, which manifests itself in the bias observed in the difference trace (SI Figure S1, bottom panel).

### Denoising on Multiple Synthetic Spectra

The power of the MCR method is that it can handle simultaneously multiple data matrices, and it is therefore ideal for coprocessing series of spectra. This is particularly relevant in the case of spectra that are acquired by varying some experimental parameter which impacts the overall sensitivity, causing some of the spectra of the series to have a significantly lower signal-to-noise ratio. This is somewhat similar to the frequency selection in non-uniformly sampled (NUS) processing as described, for instance, in refs (51−56) or the application to deconvolution of complex mixtures through multidimensional NMR.^42,54^ The synthetic test is designed to match the behavior of three spectra acquired on the same sample increasing, for example, the mixing time, therefore altering the signals intensities but not their line widths or positions.

MCR has been applied imposing the ^1^H spectrum to be common to all three experiments and allowing for variations in the ^29^Si intensities. The physical meaning of this constraint is that in all three experiments the proton source is the same and the difference resides in the efficiency of the ^1^H–^29^Si transfer as a function of the mixing time. The results are given in SI Figure S2. The improvement in the S/N ratio in the three experiments is quite large (see Table 1) and, as we had observed for the single spectrum, the shape and the relative intensities of the peaks are preserved (SI Figure S3).

**Figure 2:**
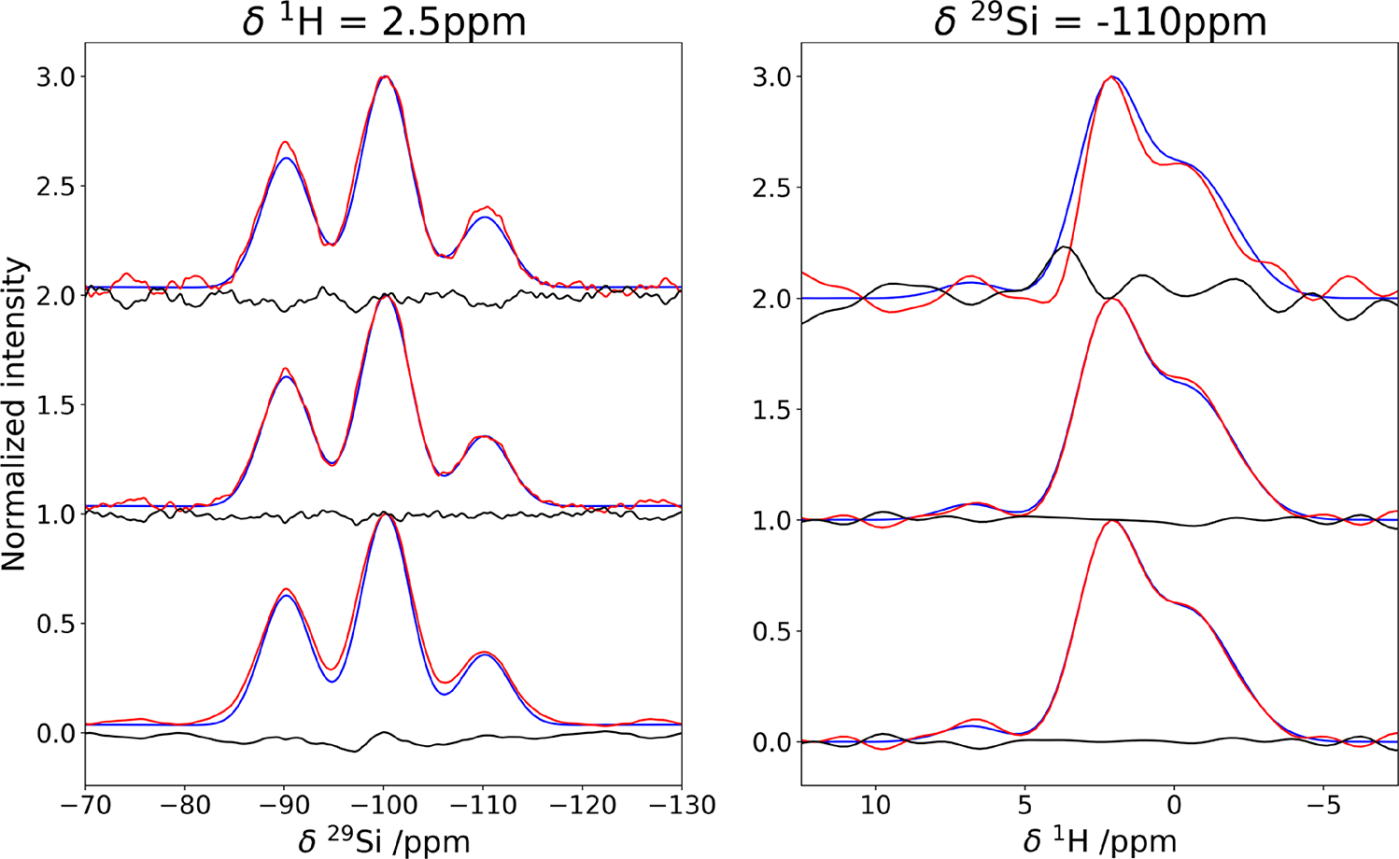
Impact of the coprocessing in the denoising of the synthetic spectrum B. In all panels, the blue trace corresponds to the noiseless spectrum. The denoising steps are listed from top to bottom: (top) initial spectrum-red trace and difference-black trace, (middle) MCR-reconstructed spectrum-red trace and difference-black trace, (bottom) MCR+Cadzow reconstructed spectrum-red trace and difference-black trace.

It is interesting to observe that, while the S/N ratio improves less for spectra B and C when they are coprocessed with A, the S/N ratio of A is largely improved when the spectra are coprocessed. At the same time, the reconstruction of all three spectra shows a higher adherence to the noiseless spectrum with respect to the individually processed spectra (Figure 2).

### The Impact of Denoising on Experimental Spectra

**Figure 3:**
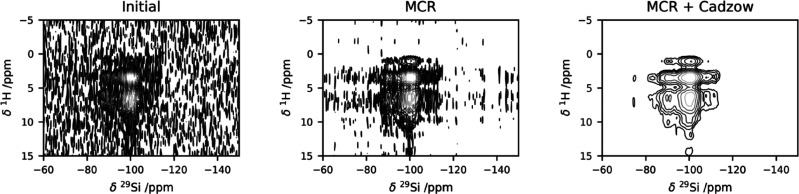
Effect of denoising on a low S/N experimental spectrum. From left to right: initial experimental spectrum, spectrum reconstructed with MCR and spectrum reconstructed with MCR after application of Cadzow denoising.

Given that the quantitative information on the spectra appears to be preserved across the denoising steps, we have applied the same procedure to experimental data sets, acquired on a silica-lysozyme composite.^19,57^ The target spectra are ^1^H–^29^Si HETCOR with Lee Goldburg homonuclear decoupling during the indirect dimension (Figure 3). Further examples are described in SI Figures S4 and S5.

**Figure 4:**
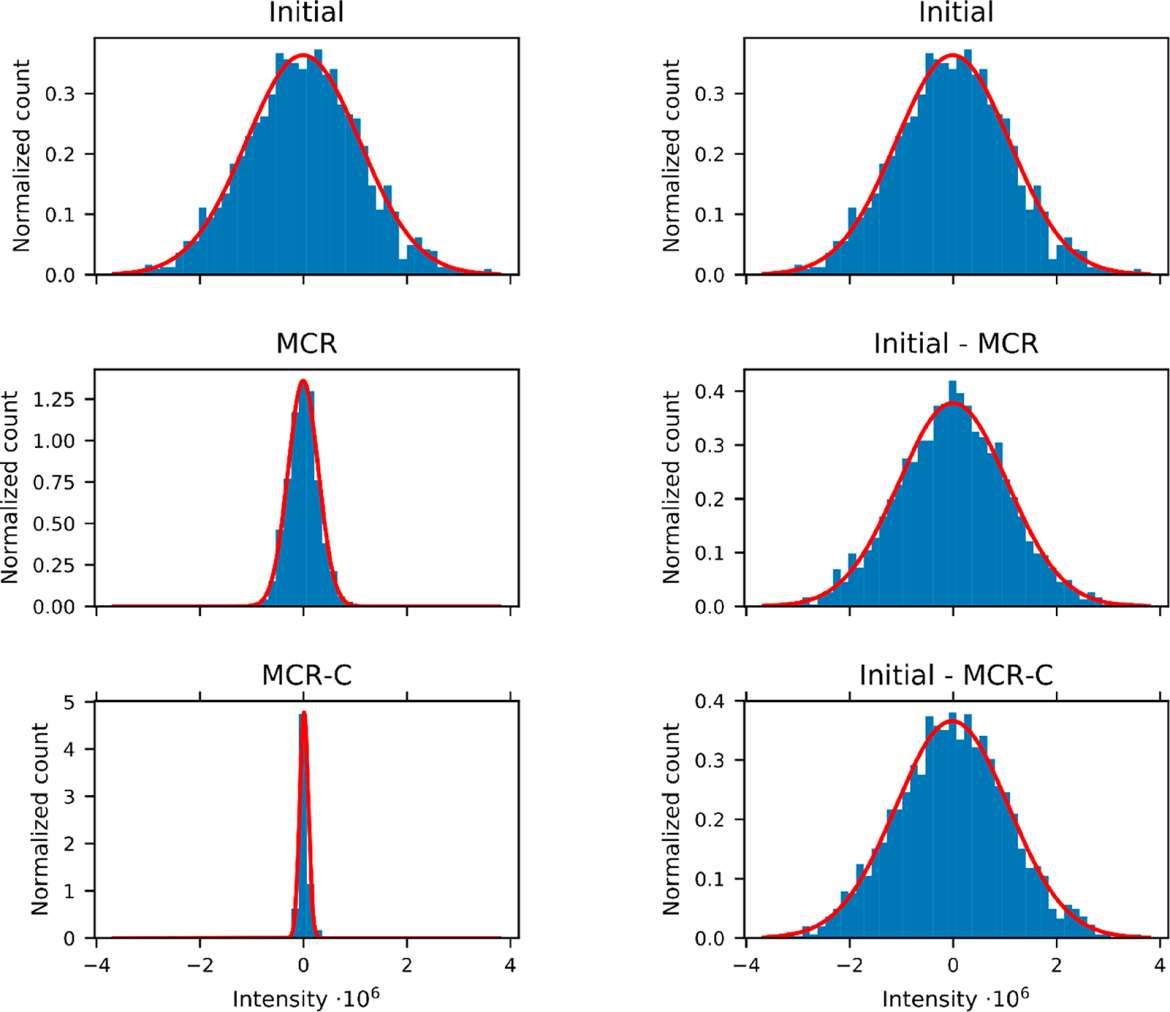
Distribution of the intensities along the points of the row of the spectrum extracted at 50 ppm, which contains only noise, representing the noise in the initial spectrum (initial) and after application of combined denoising. Left–intensity in the “signal” spectrum CS^T^; Right–intensity in the “noise” spectrum E. The red curves are the best fitting Gaussian distributions that approximates the intensities, their parameters are given in SI Table S3.

It is also extremely important to evaluate the properties of the noise, to verify that it does not change significantly during the denoising procedure. To do so, we have evaluated the difference between the initial spectrum and the processed ones and evaluated the noise distribution (Figure 4 and SI Table S3). The noise extracted from the spectra remains Gaussian. The noise distribution width is greatly decreased in the “signal” spectra (i.e.: the FT of the *CS*^*T*^ matrix), whereas the noise distribution width in the “error” spectrum (i.e.: the FT of the *E* matrix) remains the same as the noise of the initial spectrum, indicating that no part of the signal is discarded into the noise.

MCR is ideally versed toward processing of two-dimensional spectra, handling several transients at the same time, whereas other denoising methods, like Cadzow denoising, work on single transients. Therefore, one can expect that the order used to apply the different denoising schemes has an impact on the reconstruction. To test this, we have inverted the order of the processing steps (SI Figure S6) and we have found that, while the impact of MCR and Cadzow yields similar improvements in S/N ratio (from 9 to 32 and 33, respectively), MCR is able to improve the Cadzow procedure when applied first (80 for Cadzow+MCR and 99 for MCR+Cadzow).

### Combined Denoising on a Set of Experimental Spectra

**Figure 5:**
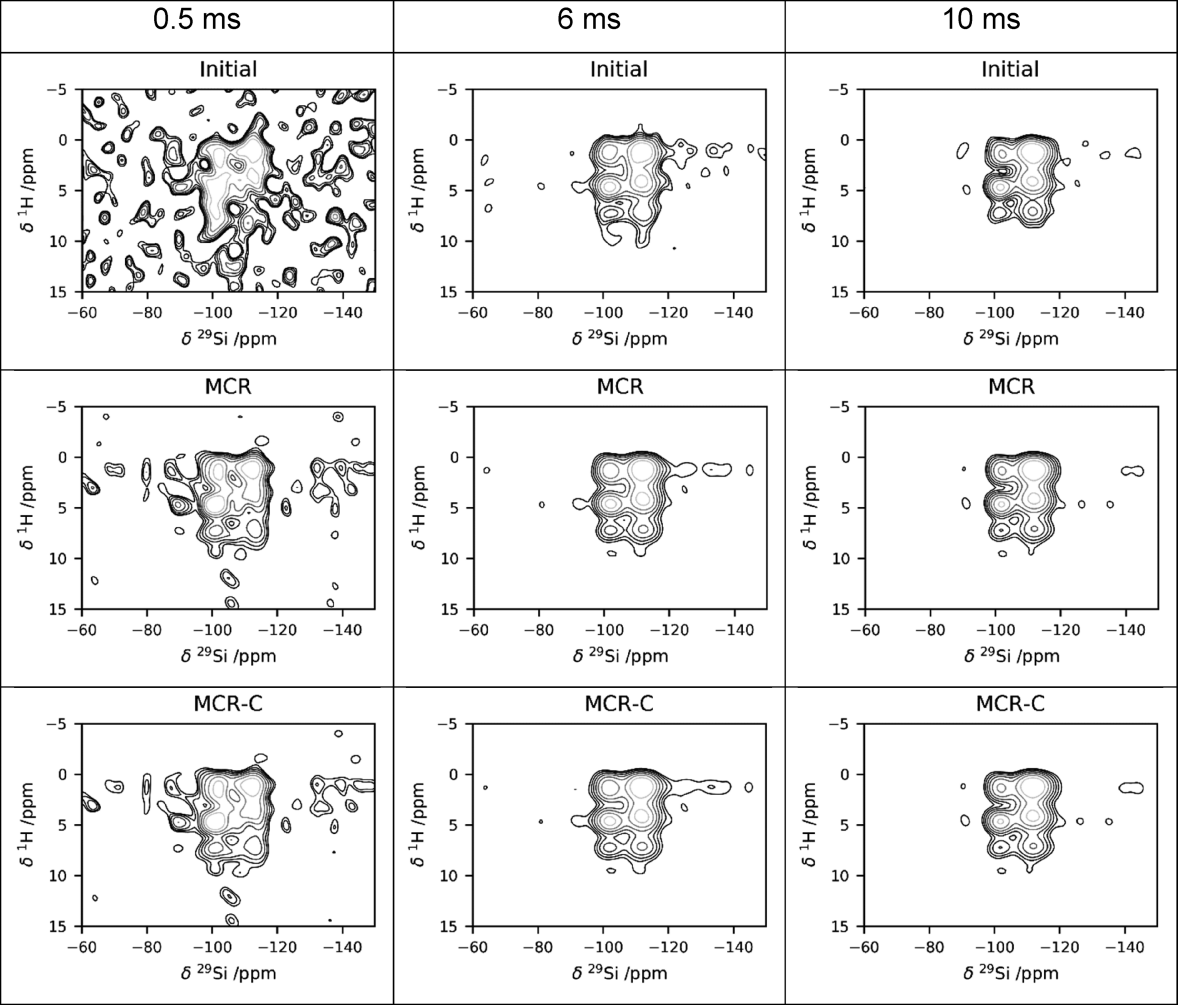
Denoising of a series of experimental spectra, acquired at different contact times: from left to right 0.5, 6, and 10 ms, respectively. (Top row) initial spectra, (central row) MCR-reconstructed spectra, (bottom row) MCR+Cadzow reconstructed spectra.

We have also applied the combined denoising to a series of ^1^H–^29^Si HETCOR spectra acquired with different contact times (0.5, 6, and 10 ms, respectively). All the echoes in the CPMG were coadded, and the resulting S/N ratio of the three spectra is 9, 42, and 60, respectively. The MCR processing increases the S/N ratio to 26, 119, and 160, and the subsequent application of Cadzow denoising further increases it to 28, 123, and 177. The results are shown in Figure 5.

## Conclusions

We have presented the use of MCR for denoising of low sensitivity solid-state NMR two-dimensional spectra. Our results demonstrate that this denoising approach preserves the quantitative information on the cross-peak intensity, yielding an improvement in the S/N ratio of around a factor of 3. Furthermore, it is robust to high levels of noise. We have also demonstrated that the intrinsic ability of MCR to coprocess multiple spectra can be used to improve the reconstruction of the spectra with the lowest S/N ratio across a series of spectra; this is particularly relevant in the case of spectra acquired on the same sample, altering some parameters in the pulse sequence, for example, varying the cross-polarization contact time across different experiments. We stress that we have applied the MCR method to time-domain data, therefore preserving the applicability of other denoising schemes, and even improving their performance, increasing the S/N ratio up to a factor of 9. Finally, our results have been obtained without imposing prior knowledge in the form of regularization of additional constraints,^42^ and thus demonstrate that this method is applicable on systems or experiments that are not described by simple forward models. Therefore, this application is not limited to ^29^Si MAS NMR, but can be applied to other nuclei of moderate or low receptivity, and also on multidimensional experiments acquired on static samples, where sensitivity is limited by the fact that the signal is spread over complicated powder patterns.^58−63^

We foresee that MCR can be generally extended to data sets of higher dimensionality and NUS sampled data.

## Dedication

We dedicate this work to the memory of Prof. Stefano Caldarelli, a brilliant mind, a very imaginative scientist, and a very dear friend. This dedication is further motivated by the fact that this work has been triggered by a discussion with him at the EMBO Workshop “Challenges for magnetic resonance in life sciences” in Principina Terra a few months before his untimely passing.
