# Supplementary material for: Multivariate Curve Resolution for 2D Solid-State NMR spectra
Source: Anal Chem. 2020 Feb 18;92(6):4451–8. doi: 10.1021/acs.analchem.9b05420 (PMC7997113; doi:10.1021/acs.analchem.9b05420)
Supplement: Supplementary file 1 — ac9b05420_si_001.pdf [file ac9b05420_si_001.pdf]

Multivariate Curve Resolution for 2D Solid-State NMR spectra

Francesco Bruno[1,2], Roberto Francischello[3,4], Giovanni Bellomo[1,2,‡], Lucia Gigli[1,2], Alessandra Flori[5], Luca Menichetti[3,5], Leonardo Tenori[1], Claudio Luchinat[1,2], Enrico Ravera[1,2]*

1. Magnetic Resonance Center (CERM), University of Florence, and Consorzio Interuniversitario Risonanze Magnetiche di Metalloproteine (CIRMMP), via L. Sacconi 6, 50019 Sesto Fiorentino, Italy

2. Department of Chemistry "Ugo Schiff", University of Florence, via della Lastruccia 3, 50019 Sesto Fiorentino, Italy

3. Institute of Clinical Physiology, National Research Council, Via G. Moruzzi, 1 56124 Pisa, Italy

4. Dipartimento di Chimica e Chimica Industriale, Università di Pisa, via G. Moruzzi 13, 56124 Pisa

5. Fondazione Regione Toscana G. Monasterio, Via G. Moruzzi 1, Pisa 56124, Italy

‡ Present address: Giovanni Bellomo, Laboratory of Clinical Neurochemistry, Neurology Clinic, University of Perugia, Piazzale Lucio Severi 1/8, 06132 Perugia (PG), Italy.

* Corresponding Author: Enrico Ravera, ravera@cerm.unifi.it

Table S1. Position, linewidth, and intensity of the cross-peaks in the synthetic spectra.

Peak position and linewidth		Intensity A	Intensity B	Intensity C
F2	F1			
-89.8 ppm, 1570 Hz	7.50 ppm, 4650 Hz	2	1	1
-89.8 ppm, 1570 Hz	4.75 ppm, 3142 Hz	1	1	1
-89.8 ppm, 1570 Hz	0.20 ppm, 3142 Hz	0	0	0.5
-99.8 ppm, 1570 Hz	7.50 ppm, 4650 Hz	3	3	3
-99.8 ppm, 1570 Hz	4.75 ppm, 3142 Hz	2	3	3
-99.8 ppm, 1570 Hz	0.20 ppm, 3142 Hz	2	3	1
-109.8 ppm, 1570 Hz	7.50 ppm, 4650 Hz	0	0	1
-109.8 ppm, 1570 Hz	4.75 ppm, 3142 Hz	0.5	2	2
-109.8 ppm, 1570 Hz	0.20 ppm, 3142 Hz	1	2	2

Table S2. Processing parameters of the 2D spectra.

Dimension	F2	F1
Nucleus	^{29}Si	^{1}H
TD	57	60
Apodization function	Sine bell	Bruker-style GM
	Power 2	LB /Hz -20
	Offset 0.35*π	GB 0.1
Zero-filling	2048	512

Table S3. Effect of the use of different number of components during the factorization. Gaussian parameters describe the properties of the noise.

**# of components**	**Spectra**	**# of iterations for optimization**	**S/N ratio**	**Gaussian parameters**	
Original	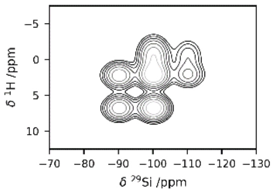	-	*42*	*μ*	*σ*
1	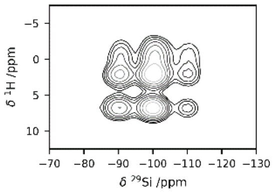	7	730	-5×10^{-7}	1.1×10^{-6}
2	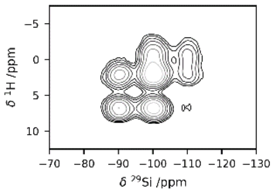	22	482	-8×10^{-7}	1.6×10^{-6}
3	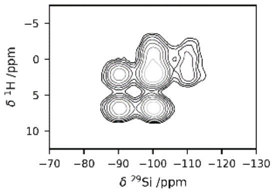	172	426	-8×10^{-7}	1.8×10^{-6}
4	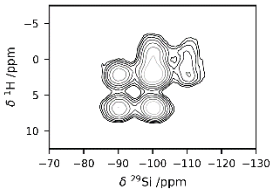	188	241	2×10^{-9}	3.5×10^{-6}
5	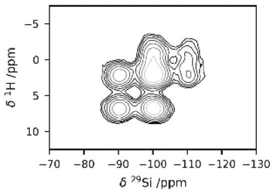	191	225	2×10^{-7}	3.7×10^{-6}
6	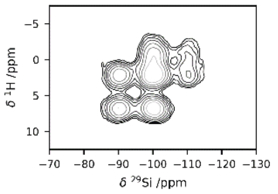	243	157	-2×10^{-8}	5.3×10^{-6}

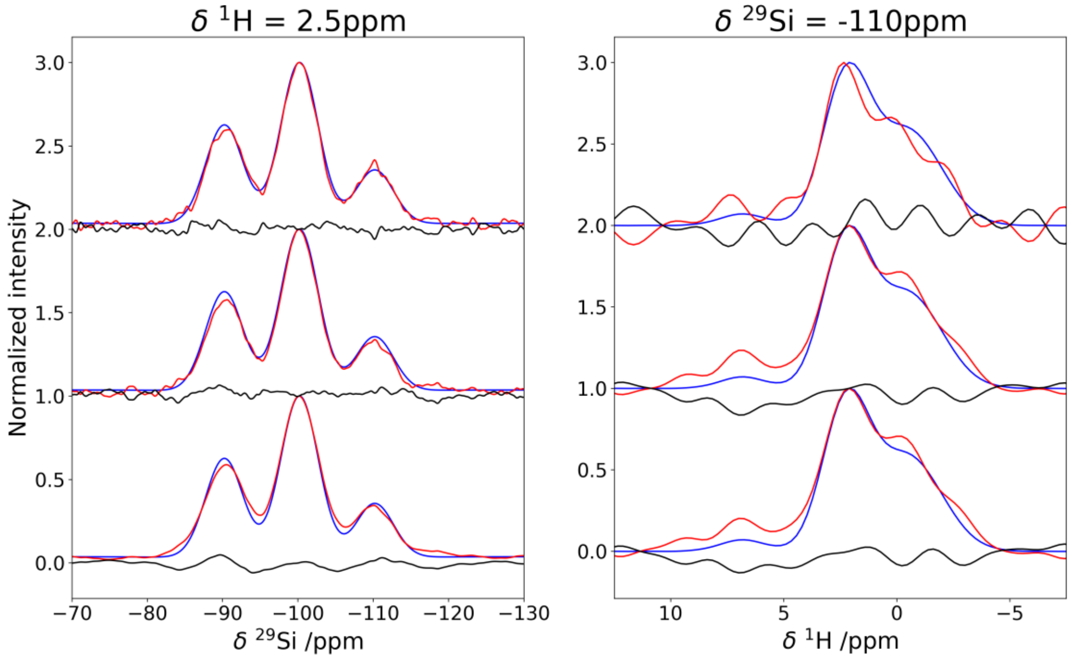

Figure S1. Impact of the denoising procedure on the synthetic spectrum, as represented through projections on the ^{1}H and ^{29}Si axes. In all panels, the blue trace corresponds to the noiseless spectrum. The denoising steps are listed from top to bottom: top) initial spectrum-red trace and difference-black trace: middle) MCR-reconstructed spectrum-red trace and difference-black trace: bottom) MCR+Cadzow reconstructed spectrum-red trace and difference-black trace. Only noise should be present if the reconstruction was perfect. In the Cadzow-reconstructed spectra, a polarization of the difference trace shows the effect of line broadening imposed by the Cadzow procedure, which gives more weight to the information arising from the first points of the FID, as well as requiring an additional exponential apodization.

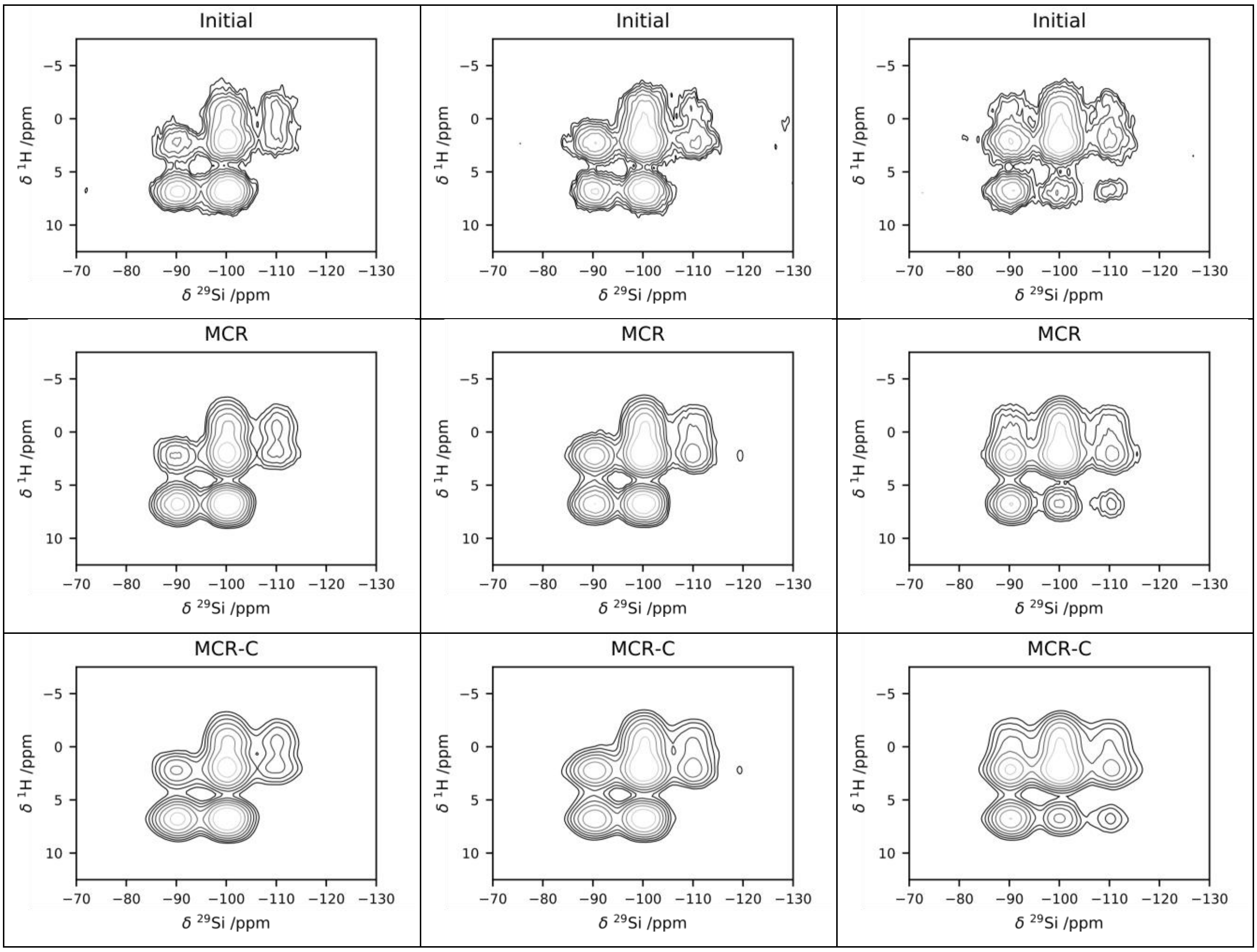

Figure S2. Effect of co-processing on a series of 2D spectra featuring different intensities of the cross-peaks (see table S1).

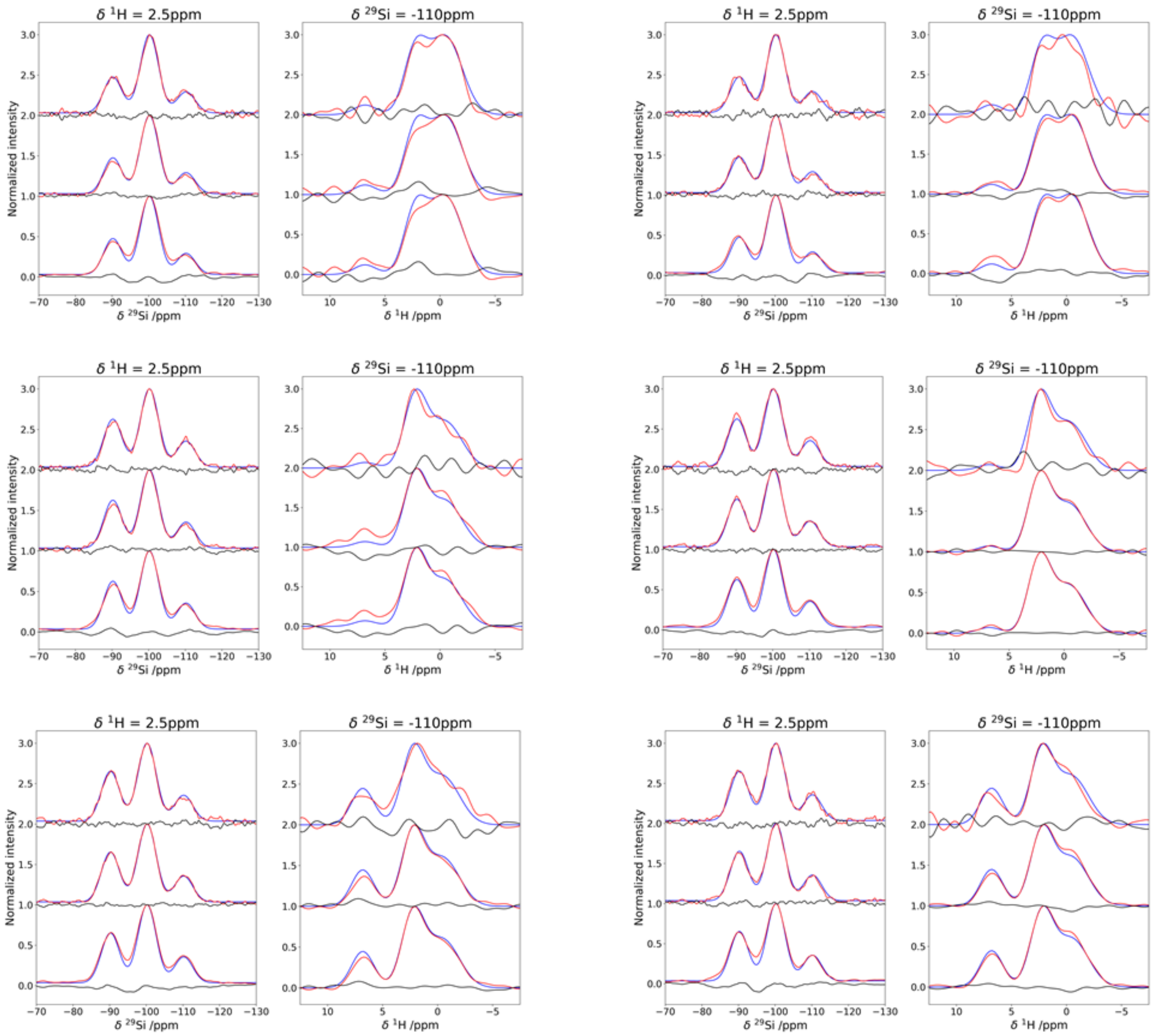

Figure S3 Impact of the denoising procedure on a series of synthetic spectra and comparison between denoising of the individual spectra (left column) and combined denoising (right column). The blue traces correspond to the noiseless spectra, the red ones to the spectra with added noise, and black ones are the difference between the noiseless spectra and the reconstruction. The denoising steps are listed from top to bottom: top) initial spectrum, middle) MCR-reconstructed spectrum, bottom) MCR+Cadzow reconstructed spectrum.

Denoising of experimental spectra with different noise levels

The HETCOR spectrum was also acquired with CPMG echo train acquisition in the direct dimension. All the echoes in the CPMG were co-added, and the resulting S/N ratio is 60. The MCR processing increases the S/N ratio to 123 and the subsequent application of Cadzow denoising further increases it to 130. The results are shown in figure S4.

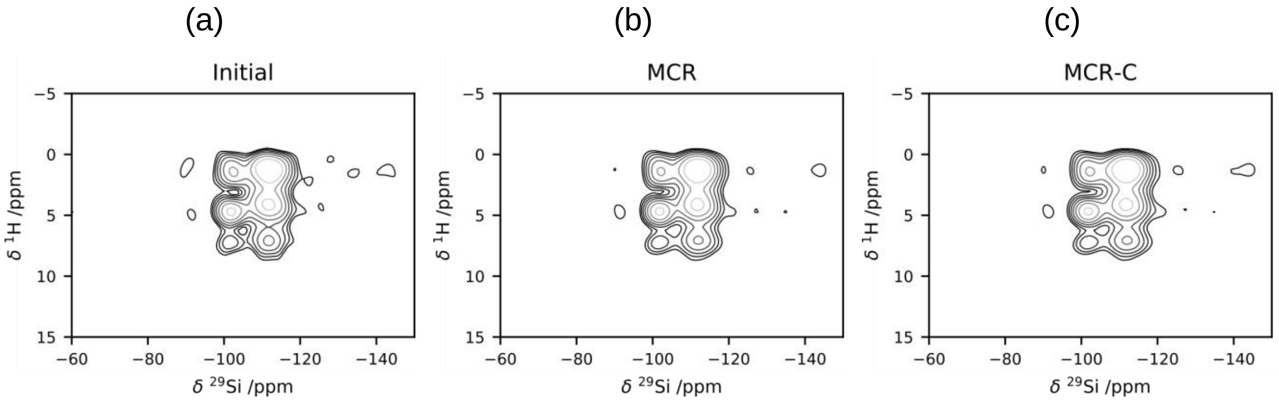

Figure S4. Denoising of experimental spectrum: (a) initial spectrum; (b) MCR-reconstructed spectrum; (c) MCR+Cadzow reconstructed spectrum.

For comparison of the denoising performance, we also selected one of the later echoes, for which the S/N ratio is only 10. The S/N ratio after MCR is 27 and it does not increase upon the application of Cadzow denoising (Figure S5).

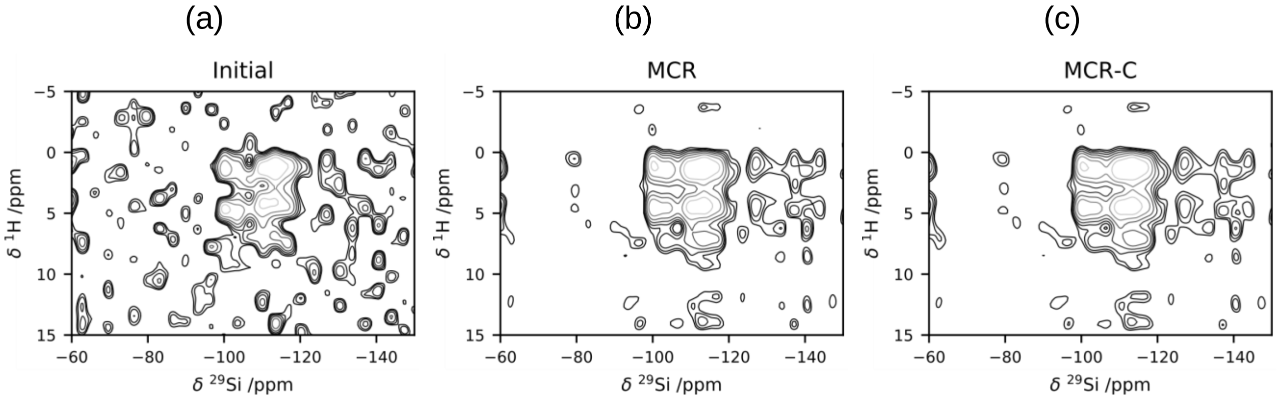

Figure S5. Denoising of experimental spectrum with a lower initial S/N ratio: (a) initial spectrum; (b) MCR-reconstructed spectrum; (c) MCR+Cadzow reconstructed spectrum.

Table S4. Properties of the noise of the spectra in figure 5 of the main text, described as a Gaussian distributions as in figure 6 of the main text.

Spectrum	D	
	μ=-0.01·10^6, σ=1.10·10^6	
Spectrum	CS^T	E
MCR	μ=-0.01·10^6, σ=0.29·10^6	μ=-0.002·10^6, σ=1.05·10^6
MCR + Cadzow	μ=0.01·10^6, σ=0.08·10^6	μ=-0.02·10^6, σ=1.09·10^6

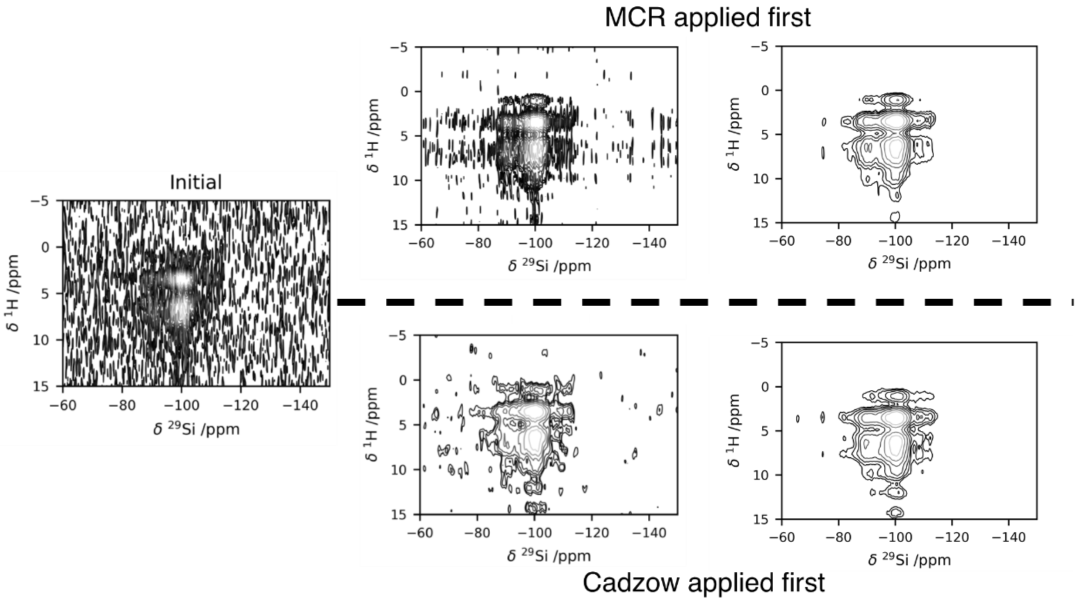

Figure S6: The effect of changing the order of the denoising steps the same low S/N experimental spectrum as figure 5.
